# Transporters Involved in Root Nitrate Uptake and Sensing by *Arabidopsis*

**DOI:** 10.3389/fpls.2016.01391

**Published:** 2016-09-21

**Authors:** Mélanie Noguero, Benoît Lacombe

**Affiliations:** Laboratoire de Biochimie et Physiologie Moléculaire des Plantes, Institut de Biologie Intégrative des Plantes “Claude Grignon”, UMR CNRS/INRA/SupAgro/UMMontpellier, France

**Keywords:** nutrient sensing, transporters, nitrates, development, plants

## Abstract

Most plants use nitrate (NO_3_^-^) as their major nitrogen (N) source. The NO_3_^-^ uptake capacity of a plant is determined by three interdependent factors that are sensitive to NO_3_^-^ availability: (i) the functional properties of the transporters in roots that contribute to the acquisition of NO_3_^-^ from the external medium, (ii) the density of functional transporters at the plasma membrane of root cells, and (iii) the surface and architecture of the root system. The identification of factors that regulate the NO_3_^-^-sensing systems is important for both fundamental and applied science, because these factors control the capacity of plants to use the available NO_3_^-^, a process known as the “nitrate use efficiency.” The molecular component of the transporters involved in uptake and sensing mechanism in *Arabidopsis* roots are presented and their relative contribution discussed.

## Introduction

Nitrate (NO_3_^-^) is an essential source of nitrogen for plant development and metabolism. With ammonium (NH_4_^+^) and urea (CO(NH_2_)_2_), it is the most used fertilizer in agriculture as a source of nitrogen (N) (Kiba and Krapp, 2016). But, beside its role as a nutrient, nitrate is also a signal molecule which is involved in the control of many physiological processes, plant growth and crop yield (Crawford, 1995; Krapp et al., 2014; Vidal et al., 2014). For example, as other nutrients like phosphate or ammonium (Drew et al., 1973; Drew and Saker, 1975), nitrate participates in the regulation of lateral roots development and architecture (Remans et al., 2006a; Walch-Liu et al., 2006b; Krouk et al., 2010a; Ruffel et al., 2011, 2014, 2016), leaf development (Chiu et al., 2004), flowering induction (Castro Marin et al., 2011) and seed dormancy (Alboresi et al., 2005). As a signal molecule, nitrate can induce the expression of a number of genes implicated in nitrate transport and assimilation (Wang et al., 2004; Bi et al., 2007; Krouk et al., 2011; Medici and Krouk, 2014).

Nitrate is often a limited resource and its accessibility is modified in both time and space, therefore plants must adapt nitrate inputs to the needs and availability in the soil. Nitrate can be assimilated in the roots or translocated to aerial organs *via* the xylem. Nitrate is then reduced to nitrite by nitrate reductase (NR) and further to ammonium by nitrite reductase (NiR) before incorporation in amino acids (Stitt, 1999). Otherwise, nitrate could also be stored, mainly in the vacuoles where concentration could vary from 5 mM to 75 mM (measured in roots of barley seedlings; Miller and Smith, 2008), in roots or shoots for further remobilization when nitrate availability became scarce.

To maximize uptake efficiency in a wide range of external nitrate concentration, plants own transport system with different properties to adjust nitrate uptake capacity (Miller et al., 2007); two types of transport systems known as Low Affinity Transport Systems (LATS) and High Affinity Transport Systems (HATS). The LATS allows transport in high (> 0.5 mM) external nitrate concentration whereas the HATS provide a capacity for nitrate absorption at low (< 0.5 mM) external nitrate concentrations. Within each of these transport systems both constitutive (c) and inducible (i) forms co-exist. Expression of these four kinds of transport systems is essential for an efficient uptake, and expression could be constitutive or inducible function of the external nitrate concentration perceived.

**FIGURE 1:**
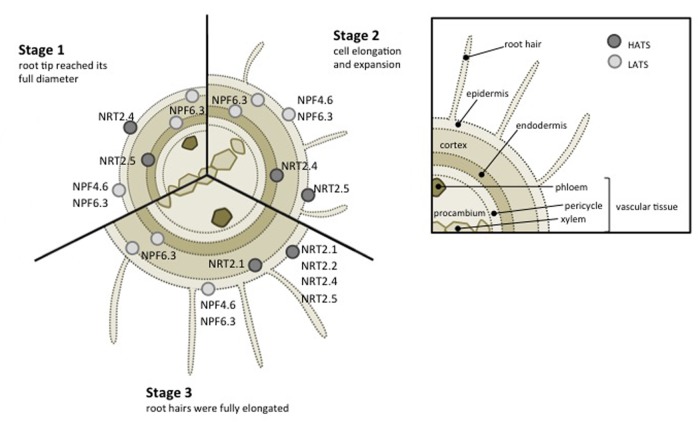
**Schematic representation of low- (light gray dots) and high- (dark gray dots) affinity nitrate transporters expressed in *Arabidopsis* root.** Three stages of root developement have been represented according to Birnbaum et al. (2003).

Four families of transporters participate in nitrate uptake, distribution or storage: NITRATE TRANSPORTER 2 (NRT2) transporters (Orsel et al., 2002; Krapp et al., 2014), NITRATE TRANSPORTER 1/PEPTIDE TRANSPORTER FAMILY (NPF) transporters (Léran et al., 2014), CHLORIDE CHANNEL FAMILY (CLC) transporters (Barbier-Brygoo et al., 2011) and SLOW ANION ASSOCIATED CHANNEL HOMOLOG (SLAC/SLAH) (Negi et al., 2008). Because roots constitute the main organ where exchange between plant and its environment take place, this review focuses on transporters identified in *Arabidopsis* root plasma membrane and contributing to nitrate uptake from the soil (**Figure 1**). Transporters from the NPF and NRT2 families are involved and interestingly some of these transporters are also involved in nitrate sensing.

## High-Affinity Nitrate Transporter: NRT2 Family

Seven members have been described in the NRT2 family in *Arabidopsis* and characterized as high-affinity nitrate transporters (Krapp et al., 2014). The affinity for nitrate of these transporters is in the range of microM, saturable around 0.2–0.5 milliM. The role in nitrate influx in the root has been demonstrated for four of them: NRT2.1, NRT2.2, NRT2.4, and NRT2.5 (Orsel et al., 2002; Kiba et al., 2012; Lezhneva et al., 2014; Kiba and Krapp, 2016).

NRT2.1 expression is found in the epidermal and cortical cells of mature roots (**Figure 1**) (Wirth et al., 2007) and constitutes the most studied transporter in NRT2 family. *NRT2.1* expression is dependent to nitrate availability and seems to be regulated by different conditions: (i) induced by nitrate (Filleur and Daniel-Vedele, 1999) and as a consequence the representative of the iHATS (inducible high affinity transport system, see Introduction), (ii) repressed in high nitrate or nitrogen concentrations (Crawford, 1995), (iii) briefly expressed in response to nitrate deficiency (Crawford and Glass, 1998; Cerezo et al., 2001). Same regulation was observed in maize (Sorgona et al., 2011). *ZmNRT2.1* expression along the maize primary root increases after 4 h of nitrate treatment coordinated with elevation of nitrate uptake rate. These results reflect an important role of NRT2.1 transporter in the regulation of nitrate fluxes in roots. Water status is affected in *nrt2.1* mutant, suggesting that NRT2.1 also supports root hydraulic conductivity (Li et al., 2016).

Despite its low expression level, *NRT2.2* expression seems to follow the same regulation (i.e., in response to nitrate availability). Moreover, *NRT2.1* and *NRT2.2* genes are very close in genomic region of chromosome 1 (AGI NRT2.1: At1g08090 and NRT2.2: At1g08100). *atnrt2* KO mutants have been obtained for both transporters NRT2.1 and NRT2.2, and are affected in the high affinity nitrate transport activity, but not in the low affinity. However, in this *atnrt2* mutant, the deletion of both transporters did not result in the complete disappearance of the nitrate uptake ability in low nitrate concentration, and besides a low affinity transport activity, a residual high affinity nitrate transport response was observed, probably due to the presence of constitutive high affinity nitrate transporters (Cerezo et al., 2001; Miller et al., 2007). In maize, an analysis of several NRT2 genes expression in response to nitrate suggests that *ZmNRT2.1* and *ZmNRT2.2* are the main genes controlling high-affinity nitrate uptake (Garnett et al., 2013).

Another NRT2 family transporter, NRT2.4, has been identified to participate to nitrate transport in roots, additionally to its contribution to nitrate distribution to shoot. Although a low expression level compared to NRT2.1, NRT2.4 is expressed in the epidermis of the lateral root (**Figure 1**; also expressed in leaves) and is involved in nitrate uptake at very low nitrate concentration. Interestingly, nitrate perception could control expression of NRT2.4, induced in long-term starvation (Kiba et al., 2012). At low external nitrate concentration (25 μM), *nrt2.1/nrt2.2* double mutant is deficient for high affinity nitrate uptake and nitrate transport is severely affected but still occurs. NRT2.4 contribute to maintain a minimal transport activity in this mutant. Kiba et al. (2012) have demonstrated that overexpression of NRT2.4 in the *nrt2.1/nrt2.2* double mutant partially restore nitrate uptake activity. However NRT2.4 is not the only one to provide this transport activity because *nrt2.1/nrt2.2/nrt2.4* triple mutant keep its ability to transport nitrate (7% relative to WT) at very low concentration (20 μM nitrate), suggesting existence of others high affinity transporters. This role is partly shared by NRT2.5 as quadruple mutant *nrt2.1/nrt2.2/nrt2.4*/*nrt2.5* has a stronger reduced high affinity nitrate transport (3% relative to WT; Lezhneva et al., 2014). Interestingly, added NRT2.4 loss of function to *nrt2.1/nrt2.2* double mutant impact much more seedling growth under N deficiency than NRT2.5. Moreover, NRT2.4 and NRT2.5 transcripts increase during N limitation, suggesting significant implication of both in root nitrate uptake (Lezhneva et al., 2014).

Interaction with a small protein NAR2/NRT3 seems to be essential for nitrate uptake of some of the NRT2 (Orsel et al., 2006; Li et al., 2007). Two members constitute NAR2 family in *Arabidopsis*, but only NAR2.1/NRT3.1 is necessary for nitrate transport activity. Indeed, co-expression in oocytes of NAR2.1/NRT3.1 and all (expect NRT2.7) NRT2 members demonstrate the positive effect of NAR2.1 on NRT2 dependent nitrate uptake (Kotur et al., 2012). Moreover, these NRT2 family members interact with NAR2.1 in yeast two hybrids (Kotur et al., 2012). The effect of NAR2.1/NRT3.1 on NRT2.4 nitrate transport activity in xenopus oocyte is yet fully understood (Kiba et al., 2012). Besides contribution to nitrate transport, NAR2.1 seems to be implicated in NRT2.1 localization or stabilization at the plasma membrane (Wirth et al., 2007). NAR contribution to nitrate transport is also observed in others species, like barley, maize or rice. Measurements of the ^15^N-nitrate enrichment in oocytes showed that co-injection of HvNRT2.1 and HvNAR2.3 was able to provide significant nitrate uptake (Tong et al., 2005). Lupini et al. (2016) show that ZmNAR2.1 has an important implication in ZmNRT2.1 expression and localization along root axis correlated with nitrate influxes (Lupini et al., 2016). In rice, the expression of the Os*NRT2.1, OsNRT2.2*, and *OsNRT2.3a* genes seems to be regulated by *OsNAR2.1*, itself transcriptionally induced by nitrate (Yan et al., 2011).

## Low-Affinity Nitrate Transporter: NPF Family

In higher plants, NPF family includes a large number of genes, divided into eight subfamilies, and able to transport diversified substrates. However, no substrate selectivity correlates with sequence homology, and consequently with each subfamily (Léran et al., 2014). In *Arabidopsis*, NPF family consists of 53 members mainly characterized as low affinity transporters. Herein, NPF6.3/NRT1.1/CHL1 was firstly identified as low affinity nitrate transporter (Tsay et al., 1993). *NPF6.3* is expressed in several root tissues: epidermis, cortex and endodermis (**Figure 1**; also expressed in young leaf and flower buds) (Huang et al., 1996; Guo et al., 2002; Remans et al., 2006a). Consequently, NPF6.3 allows nitrate uptake from soil (Crawford, 1995; Munos et al., 2004) and is also implicated in nitrate translocation to aerials part (Boursiac et al., 2013; Léran et al., 2013). NPF6.3 reveals also an ability to switch to high-affinity activity in low nitrate conditions (Liu et al., 1999; Liu and Tsay, 2003; Ho et al., 2009). When phosphorylated, Thr 101 confers to NPF6.3 high affinity nitrate transport behavior whereas non-phosphorylated NPF6.3 acts as low-affinity transporter. The protein complex CIPK23-CBL9 (CIPK: CBL-Interacting Protein Kinase, CBL: Calcineurin-B like Protein) is implicated in the dual affinity transition changes thanks to its ability to phosphorylate Thr 101 residue. In low nitrate conditions, CIPK23-CBL9 phosphorylates NPF6.3 Thr 101 residue and promotes NPF6.3 high affinity nitrate transport (Liu and Tsay, 2003; Ho et al., 2009). Other proteins, namely ABI1 and ABI2 belonging to protein phosphatase 2C family from the clade A, contribute to NPF6.3 activity in xenopus oocytes (Léran et al., 2015). *In planta* experiments demonstrate that only ABI2 has a regulatory role on NPF6.3-dependent nitrate transport. This protein was able to modulate transport activity by preventing phosphorylation of CIPK23-CBL1 complex (Léran et al., 2015).

Functional characterization and regulation of NPF6.3 transporters is still ongoing and many residues seem to be implicated in nitrate transport ability. Recently, crystallization studies of NPF6.3 reveal a crucial role of His 356 residue in nitrate substrate binding and transport. In the presence of nitrate, authors show that NPF6.3 H356A variant loses its nitrate transport capacity compared to wild type NPF6.3 protein (Parker and Newstead, 2014; Sun et al., 2014).

NPF4.6/NRT1.2/AIT1, another nitrate transporter belonging to NPF family is involved in soil nitrate uptake. As for NPF6.3, NPF4.6 is also expressed in root epidermis (**Figure 1**), however, NPF4.6 display a constitutive expression and activity is restricted to low affinity nitrate transport (Liu et al., 1999). NPF4.6 is also an ABA transporter implicated in seed dormancy and transpiration (Kanno et al., 2012).

NPF2.7/NAXT1 is another member of the NPF family expressed the cortex of mature roots (**Figure 1**) and implicated in root nitrate uptake in *Arabidopsis*. However, it seems to be mainly involved in nitrate eﬄux to the external media and consequently participate in nitrate homeostasis (Segonzac et al., 2007). As for others NPFs (i.e., NPF7.2, NPF7.3, and NPF2.3) expressed in *Arabidopsis* roots and characterized to date, their role consist mainly to nitrate translocation to xylem tissue for distribution to aerial parts (Lin et al., 2008, Li et al., 2010; Taochy et al., 2015).

## Nitrate Sensing by Transporters

Although it constitutes an essential nutrient, nitrate is also known to be a signaling molecule involved in many physiological processes including gene regulation (Wang et al., 2004) and root development (Walch-Liu et al., 2006a). Within the different proteins involved in nitrate sensing (Medici and Krouk, 2014), two transporters described above, namely NRT2.1 and NPF6.3/NRT1.1, are key ones.

### NRT2.1

Formation of lateral root is strongly affected by environmental condition and nutrient signals as nitrate are essential to influenced root architecture. Beside its role in high-affinity nitrate transport, NRT2.1 is involved in nitrate-dependent lateral root initiation (LRI). With genetic screen using the repression of LRI in seedlings growing on high sucrose/low nitrogen conditions, a *lin1* mutant has been isolated for its ability to initiate a large number of lateral roots (Malamy and Ryan, 2001). Further characterization of *lin1* mutant indicates a point mutation in NRT2.1 gene that leads to G119R substitution in the protein sequence is responsible for *lin1* phenotype (Little et al., 2005). Loss of function *lin1* mutant as well as three others *nrt2.1* mutants (*nrt2.1-1*, *nrt2.1-2*, and *nrt2.1-3*) exhibit nitrate-independent increases lateral root formation suggesting that NRT2.1 is implicated in LRI repression under specific conditions (i.e., high sucrose/low nitrate). Nitrate influx in *lin1* (as *nrt2.1* KO) mutant is significantly reduced compared to WT, indicating that nitrate uptake and/or accumulation could be correlated to inhibition of lateral root primordia (Little et al., 2005; Remans et al., 2006b). However, LRI is not only regulated by direct sensing of the external nitrate concentration and could be influenced by long-term nitrate growth condition. Thus, decrease in nitrate availability [seedling transferred from high (10 mM) to low (0.5 mM) nitrate media] seems to stimulate both lateral root length and LRI in WT plants (Remans et al., 2006b).

These results indicate that NRT2.1 deals with nitrate transporter activity independently from its nitrate sensors activity in the root development control. Nitrate effect on root development not only originates from current nitrate transport but also from nitrate already available from the plant.

### NPF6.3/NRT1.1/CHL1

In addition to its nitrate transport activity (Tsay et al., 1993), NPF6.3/NRT1.1 functions as a nitrate sensor and is able to promote physiological response in the control of root system architecture and to modulate the expression level of many gens implicated in nitrate signaling pathway (Wang et al., 2004; Krouk et al., 2009, 2010b; Medici and Krouk, 2014; Medici et al., 2015). The NPF6.3 dependent regulation of NRT2.1 expression has been studied and is modulated according to nitrate concentration. Fast induction of NRT2.1 expression is observed in response to brief exposure to nitrate concentration (Ho et al., 2009; Bouguyon et al., 2015) whereas NRT2.1 is down regulated in long-term high nitrate supply (Munos et al., 2004; Krouk et al., 2006). Another nitrate dependent phenotype, lateral root development, is also NPF6.3/NRT1.1-dependent (Bouguyon et al., 2016). Thus in low nitrate condition, NPF6.3 acts as a repressor of lateral root primordia and it became an activator of root branching in response to nitrate supply (Remans et al., 2006a; Krouk et al., 2010a; Mounier et al., 2014).

The definitive proof of the role of NPF6.3/NRT1.1 in nitrate sensing has been given by the study of *chl1-9*, NPF6.3/NRT1.1-P492L (Ho et al., 2009). Although a normal level of transcript and protein expression, nitrate transport activity was suppressed in *chl1-9* knockout mutant, demonstrating that the Pro 492 residue is essential for nitrate uptake. Interestingly, this mutant is still able to induce NPF6.3/NRT1.1-dependent gene expression measured through NRT2.1 expression level, indicating that PNR is not affected in this mutant. This specific mutation demonstrates that the roles of NPF6.3 in transport and signaling belong to independent regulations (Ho et al., 2009; Bouguyon et al., 2015).

NPF6.3 ability to modulate lateral root development is mediated by its auxin transport capacity (Krouk et al., 2010a; Bouguyon et al., 2015). Correlations have been suggested between NPF6.3 auxin transport ability and nitrate sensing and signaling. Indeed, P492L and T101A point mutations decrease auxin transport capacity and plants expressing these mutants (i.e., *chl1-9* and NPF6.3-T101A) are affected in nitrate-dependent lateral root development, suggesting that phosphorylated form of NPF6.3 could be responsible for NPF6.3 dependent regulation of both nitrate and auxin transport and consequently for lateral root development (Bouguyon et al., 2015).

Finally, the NPF6.3/NRT1.1 regulators, the kinase CIPK23 and the phosphatase ABI2, are also involved in nitrate sensing (Ho et al., 2009; Léran et al., 2015): CIPK23 being a negative regulator of NPF6.3/NRT1.1 by phosphorylation, and then a repressor of PNR whereas ABI2 being a positive regulator of the negative regulator complex (CIPK23/CBL1). Recently, the *NRG2* (Nitrate Regulatory Gene) gene has been described to impact nitrate signaling *via* regulation of NPF6.3 in roots (and NPF7.2 in leaves). *nrg2* mutant grown in nitrate media supplemented with ammonium shows a lower expression of NPF6.3 expression in roots as well as down regulation of many nitrate responsive genes. Consequently, theses mutants display a defect in nitrate accumulation on roots (Xu et al., 2016).

## Conclusion

Two transporters, NRT2.1 and NPF6.3/NRT1.1, as well as their protein partners (NAR2, CBL1, CBL9, CIPK23, ABI2) are involved in nitrate transport and sensing. Although theses nitrate transporters are among the most studied, there are still some missing parts to better interpret the transport regulation and the modulation of root architecture, and as well the adjacent mechanisms responsible for optimization of nitrate use efficiency (NUE; O’Brien et al., 2016). Plant nitrate uptake constitutes an important trait to take into account to improve crop yield and NUE. Indeed in rice, plants overexpressing the high affinity nitrate transporter NRT2.3b have an increased nitrate uptake correlated to an improvement in growth capacity, yield and NUE (Fan et al., 2016), just like OsNRT1.1b also implicated in nitrate transport efficiency and NUE (Fan et al., 2015; Hu et al., 2015). Thus, the capacity of other NPF and NRT2 transporters to sense nitrate will be determined in further experiments and will help us to better understand how plant is able to cope with nitrate heterogeneity. Furthermore, identification of proteins belonging to the NRT2 and NPF6.3/NRT1.1 protein regulatory networks will give new insights in the nitrate sensing capacity of plants. This review deals with plasma membrane transporters, however, vacuolar transporters are involved in nitrate accumulation and consequently impacts on NUE, as observed in rapeseed for *BnNRT1.5* (Han et al., 2016). Thus, determination of molecular players and regulatory factors interacting with nitrate transporters, as well as others transporters like SLAH1 through which plants are able to balance between nitrate and chloride loading (Cubero-Font et al., 2016) constitutes a future challenge to better understand how nitrate influences plant growth and development.

## Author Contributions

All authors listed, have made substantial, direct and intellectual contribution to the work, and approved it for publication.

## Conflict of Interest Statement

The authors declare that the research was conducted in the absence of any commercial or financial relationships that could be construed as a potential conflict of interest.
